# From the Evasion of Degradation to Ubiquitin-Dependent Protein Stabilization

**DOI:** 10.3390/cells10092374

**Published:** 2021-09-09

**Authors:** Yamen Abu Ahmad, Avital Oknin-Vaisman, Eliya Bitman-Lotan, Amir Orian

**Affiliations:** Rappaport Faculty of Medicine, R-TICC, Technion-IIT, Efron St. Bat-Galim, Haifa 3109610, Israel; yamena@campus.technion.ac.il (Y.A.A.); ginjes01@gmail.com (A.O.-V.); eliyabit@technion.ac.il (E.B.-L.)

**Keywords:** ubiquitin, proteasome, degron, heterotypic-Ub chains, E3 ubiquitin ligases, degradation, protein-stabilization, degradation-resistant tumors, RNF4, STUbL, oncoproteins, cancer

## Abstract

A hallmark of cancer is dysregulated protein turnover (proteostasis), which involves pathologic ubiquitin-dependent degradation of tumor suppressor proteins, as well as increased oncoprotein stabilization. The latter is due, in part, to mutation within sequences, termed degrons, which are required for oncoprotein recognition by the substrate-recognition enzyme, E3 ubiquitin ligase. Stabilization may also result from the inactivation of the enzymatic machinery that mediates the degradation of oncoproteins. Importantly, inactivation in cancer of E3 enzymes that regulates the physiological degradation of oncoproteins, results in tumor cells that accumulate multiple active oncoproteins with prolonged half-lives, leading to the development of “degradation-resistant” cancer cells. In addition, specific sequences may enable ubiquitinated proteins to evade degradation at the 26S proteasome. While the ubiquitin-proteasome pathway was originally discovered as central for protein degradation, in cancer cells a ubiquitin-dependent protein stabilization pathway actively translates transient mitogenic signals into long-lasting protein stabilization and enhances the activity of key oncoproteins. A central enzyme in this pathway is the ubiquitin ligase RNF4. An intimate link connects protein stabilization with tumorigenesis in experimental models as well as in the clinic, suggesting that pharmacological inhibition of protein stabilization has potential for personalized medicine in cancer. In this review, we highlight old observations and recent advances in our knowledge regarding protein stabilization.

## 1. Overview

The dynamic stability of proteins, which is a fundamental concept in biology, was discovered by Rudolph Schonheimer [1]. It is, however, well known that proteomes exhibit a wide range of protein half-lives [2]. While some intracellular proteins are extremely long-lived (LLPs; [3]), many regulatory proteins degrade rapidly, exhibiting a half-life of only minutes. In this study, we relate to protein abundance/stabilization and to changes in protein half-life that are determined by regulated degradation rather than by the biophysical structural properties of the protein. A central system that mediates ATP-dependent degradation of proteins is the ubiquitin-proteasome system (UPS; [4,5,6]). The degradation rate of a given protein is not, however, fixed; it is physiologically dynamic and dramatically affected by post-translational modifications such as phosphorylation, hydroxylation, acetylation, SUMOylation, and methylation, that may directly effects degrons [7]. For example, upon immune challenge, signal-induced phosphorylation of the inhibitory protein, IκBα, converts the extremely stable protein into a rapidly degraded phospho-protein, enabling immune response [8,9].

**Figure 1:**
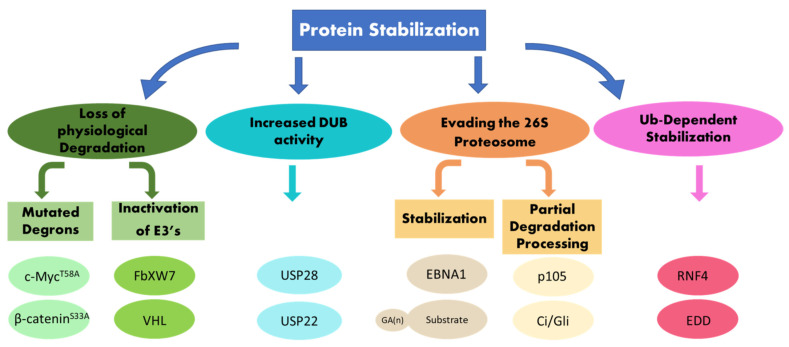
Mechanisms of protein stabilization. Schematic diagram for mechanisms resulting in protein stabilization that are discussed in this review, see text for details.

Protein stability depends on intrinsic structures within the targeted proteins, called degrons, as well as on enzymes that mediate the ubiquitination of the protein substrate. A recent systemic analysis of primary tumors established that stabilizing mutations in degrons and inactivating mutations in ubiquitin ligases (E3), which mediate the recognition of such degrons, are driving events in tumorigenesis [10]. Active stabilization, or limited proteolysis, also depends on the type of ubiquitin chains catalyzed on the protein substrates. In addition, a ubiquitin-dependent protein stabilization pathway actively stabilizes proteins in cancer. This pathway is situated upstream to and independent of the machinery involved in their degradation. A central enzyme in this pathway is the ubiquitin ligase RNF4 [11]. In this review, we address several aspects of protein stabilization. First, we discuss cases of protein stabilization that result from mutations in degrons or inactivation of the enzymes that mediate the degradation of these proteins. Second, we focus on cases of protein stabilization that stem from active ubiquitin-dependent protein stabilization, which is highly relevant to cancer (Figure 1).

## 2. Evading Recognition I: Protein Stabilization Due to Lacking or Mutated Degradation Signals

Degrons are short amino-acid sequences that determine the stability of proteins. They are divided according to their position on the protein substrate to N-terminal, C-terminal, and internal degrons. The biochemical nature of the N- and C-terminal degrons and the enzymatic pathways dedicated to the recognition thereof are discussed in detail in a recent review by Varshavsky [12], who also discovered the contribution of N-terminal degrons to protein stability in 1986 [13].

Internal degrons serve as recognition motifs for the diverse group of E3 ubiquitin ligases [14]. Internal degrons were initially thought to mediate only the docking and physical interaction between the substrate and the E3 ubiquitin ligases that catalyze their ubiquitination and subsequent degradation. However, it is now clear that these motifs also contain sequences that enable stabilizing signals [15,16,17]. Among the first degrons to be identified was the Delta motif, a stretch of 27 amino acids that is present in the c-Jun proto-oncogene and is required for its ubiquitin-dependent degradation. This motif is not present in v-Jun, the oncogenic viral protein, or in its closely related JunD, which exhibits a longer half-life [18,19].

Another example of a short recognition motif is a motif present in P53 tumor suppressor, which binds to a cleft on the surface of the oncogenic RING ubiquitin ligases HDM2 and MDM2 [20]. Targeting this interaction surface and blocking it with a group of small molecules stabilizes P53. These small molecules are currently being tested in clinical trials in tumors harboring wild-type P53 [21,22]. Moreover, MDM2 itself is stabilized by Akt-phosphorylation [23], as well as by the HECT-ubiquitin ligases, WWP1 and NEDD-4 (see review by Chenghua Li in this volume and [24,25]).

A subgroup of internal degrons is recognized by Cullin-based RING ubiquitin ligases and includes Skp-Cullin-F-box ubiquitin ligase complexes (SCF), where the F-box protein serves as a receptor subunit that binds to a phosphorylated degron via its C-terminal WD40 domain. One example of such a degron is that recognized by the F-box protein β-TRCP, DpSGXX (X) pS (where X is any amino acid). Among the substrates that contain this degron are IκBα and β-catenin [26,27,28]. To date, more than 200 substrates containing this degron have been identified [29].

In cancer, oncoprotein stabilization is the direct result of mutations within degrons. Specifically, mutations in the phosphorylated residues abolish the binding of F-box proteins that recognize phosphorylated degrons. It was observed early on that colon cancer and melanoma genomes are characterized by mutations, substituting Ser33 and Ser37 with Ala within the β-catenin degron, resulting in a stable and active nuclear co-activator protein [30,31]. Later, it was shown that the binding and ubiquitination of β-catenin by the E3 ligase β-TRCP requires phosphorylation of the β-catenin degron at these sites by GSK-β [32,33]. Remarkably, the ubiquitin ligase EDD recognizes GSK-β-phosphorylated (Ser33) β-catenin, resulting in its stabilization, nuclear accumulation, and transcriptional hyper-activation [34].

The F-Box protein FbXW7, likewise, recognizes a short phospho-degron that is present in key G1/S regulators such as Myc proteins, CycE, c-Jun, Notch-intracellular domain (N-ICD) proteins, mTor, PGC-1α, and the simian virus-encoded oncoprotein Large-T antigen [35,36,37]. In Burkitt’s lymphoma, the oncogenic mutation Thr58Ala located in the c-Myc main degron abolishes its binding to FbXW7, stabilizes c-Myc, and is a hotspot for mutations [38,39,40]. A detailed analysis of degrons in cancers is discussed in [7] and the reader is referred to a detailed description of degrons therein (Table 1 in [7]). Beyond specific examples, leveraging machine learning and using cancer datasets, Takheim et al. found that mutations affecting degrons play an important role in driving tumorigenesis [41]. The authors developed “deepDegron”, a machine learning method that predicts the impact of mutations or degron loss on protein stability, and validated these predictions for mutations in the degrons of GATA3 and PPM1D that result in stable proteins. Interestingly, in many cases, degrons are localized to intrinsically disordered regions (IDRs), and recent studies suggest that recognition, ubiquitination, and degradation take place in a unique physical environment involving phase separation [42,43].

Transient protein stabilization may also be due to sequestration or inactivation of the kinase(s) phosphorylating the degron, a phosphorylation that is essential for E3 ligase recognition. During activation of Wnt/LRP6 signaling, the kinase GSK-β which phosphorylates β-catenin, and FbXW7 substrates, such as c-Myc, are sequestered, resulting in the stabilization of β-catenin, c-Myc, and other critical tumor-promoting regulators [44].

Thus, oncoprotein stabilization may stem from evolutionary forces and driving mutations within oncoprotein degrons, generating more stable and active proteins that evade recognition by the ubiquitination machinery that promotes tumorigenesis. It should be noted, however, that the real-life (“outside-the-textbook”) biology of degrons is more complex; the true degradation rate of a given protein is impacted by other weak degrons in the same protein, as well as additional factors such as the capacity of F-Box proteins to dimerize [45].

## 3. Evading Recognition II: Protein Stabilization Due to Impaired E3 Ubiquitin Ligase Activity, and the Emergences of Degradation-Resistant Tumors

Protein stabilization also results from genetic, epigenetic, or post-translational inactivation of the ubiquitin E3 ligases that mediate the ubiquitination and subsequent proteasomal degradation of short-lived oncoproteins. E3s ligases are, therefore, the enzymes that contribute most to the specificity of the ubiquitin system. The human genome encodes ~ 800 ubiquitin ligases that are divided into subfamilies based on central domains such as HECT, RING, RBR, and Ubox [14,46]. For example, enzymes such as Mdm2 or E6-AP, which ubiquitinate the tumor suppressor proteins P53, are oncogenic [47,48,49,50]. In contrast, ligases such as FbXW7 or β-TRCP, whose activity leads to the degradation of oncoproteins, are bona fide tumor suppressors. Furthermore, ubiquitination is linked to other protein destruction pathways such as lysosomal degradation of cell surface receptors. For example, inactivation of ubiquitin ligase Cbl results in stabilization of growth factors that drive receptors such as EGFR by increasing their half-life on the cell surface, resulting in sustained oncogenic signaling [51]. The reader is referred to excellent recent reviews of the biology of ubiquitin ligases in cancer [46,52]. Figure 2 in [46] presents an elegant scheme of ligases that are inactivated in cancer, as well as their substrates and relationship to the hallmark cancer.

One consequence of E3 inactivation is the development of degradation-resistant tumor cells that accumulate multiple, stabilized, and active oncoproteins. Loss of FbXW7, the receptor subunit within SCF^FbXW7^, results in stabilization of multiple oncoproteins that all drive G1/S progression (e.g., CycE, c-Myc, NICD, c-Jun). The collective stabilization of multiple oncogenic drivers is a frequent phenomenon that is also observed for other ligases; thus, inactivation of Cbl, results in stabilization of multiple cell surface receptors [53]. Another case is the stabilization of substrates of the VHL ligase complex beyond its classical substrate, the hydroxylated hypoxia-induced factor HIF [54]. Thus, the development of tumors due to loss of E3 ligase activity gives the transformed cell a dramatic evolutionary advantage over the surrounding non-transformed cells. It is a challenging entity, both clinically and for pharmacological targeting, unlike the case of cancer that arises from the stabilization of a single oncoprotein.

## 4. Evading the Proteasome: Stabilization and Limited Proteolysis Signals

Ubiquitinated proteins are recognized, unfolded, and degraded into short peptides by the 26S proteasome, a large ATP-dependent multi-subunit protease complex [55]. Nevertheless, short amino acid sequences or domains within proteins completely inhibit proteasomal degradation or serve as ”stop” signals, enforcing limited proteolysis/processing. One such inhibitory sequence is the Gly-Ala repeat (GAr), which is present in the Epstein--Barr virus (EBV) protein EBNA-1 as a 398-residue-long internal stretch [56]. EBNA-1 is expressed during latent EBV infection and is commonly detected in all chronic EBV carriers. It is the only viral protein regularly detected in tumors associated with EBV. EBNA1 does not stimulate a cytotoxic T-cell response as EBNA1-derived peptides are not generated nor presented by MHC-I antigen-presenting cells, a process that is mediated by the proteasome [57]. Wild-type EBNA1 is stable, and deletion mutants of EBNA-1 lacking the GAr degrade rapidly and are presented on the surface of MHC-Class I molecules, inducing T-cell activity. Moreover, addition of the GAr to the well-degraded and presented EBNA-4 results in a stable protein that is not presented by MHC Class I molecules [58]. Remarkably, the GAr is, in some cases, a transferable “stop” signal; it can stabilize and protect P53 from Mdm2- or E6AP-mediated degradation as well as chimeric proteins in yeast [59,60]. The exact mode of action of the GAr is unclear, and even short GA repeats are sufficient to halt degradation. Nevertheless, the presence of a strong degron was able to overcome even a long GAr (>200 a.a.) [61]. While these repeats do not interfere with the ubiquitination of the substrate, they likely reduce the residence time of the substrate on the proteasome and may impair substrate unfolding [62].

GArs serve not only as protein stabilizers but also as internal barriers leading to limited proteolysis/processing and protein aggregation. A well-studied case is the generation of the NF-κB subunit p50 by proteolytic processing from its NFκB1 p105 precursor protein. A 30 a.a.-long glycine-rich region (GRR) is required for the limited processing of p105 and deletion of the GRR results in complete degradation of the p105 precursor. In this case, a short GAGAGA sequence is sufficient for generating the p50 subunit, but with lower yield, likely inducing the full degradation of the precursor. The GRR also confers stability to the newly formed p50 subunits [63,64]. A GRR is also present and required for the increased stability and aggregation of the 43 kDa TAR DNA-binding protein (TDP-43), and the short peptide related to the GRR is capable of forming aggregate-prone fibrils [65,66].

Another case of ubiquitin-dependent processing is the generation of the transcriptional repressors Cubitus interruptus (Ci)/Gli proteins that function downstream hedgehog signaling. The Ci75 repressor protein is generated by processing its precursor Ci155 [67], but the structural “stop” signal limiting Ci proteolysis is not a GAr/GRR but rather part of a zinc-finger domain [68]. In the case of Ci, β-TRCP is the ubiquitin ligase involved in processing, while ubiquitination by HIB, a BTB E3 ligase, leads to the complete degradation of Ci155 [69,70].

Interestingly, in the case of p105, limited proteolysis is mediated by the E3 KPC, while the ubiquitin ligase β-TRCP mediates the complete degradation of p105 [71,72,73,74]. In both cases, it is still not clear mechanistically how ubiquitination by distinct ligases has differential effects on the same protein. It is possible that in addition to the structural barrier, the type of ubiquitin chains generated by the E2/E3, potentially together with ancillary factors at the proteasome level, may determine the exact outcome: complete destruction or limited proteolysis.

Another domain that confers protein stabilization in the vicinity of the proteasome is the ubiquitin-associated 2 domain (UBA2) of Rad23, which is a bona fide LLP. Rad23 serves as a ubiquitin receptor that delivers ubiquitinated proteins to the proteasome and the UBA2 domain protects Rad23 from proteasomal degradation [75,76]. It is possible that UBA2 functions as a tightly folded domain that prevents the unfolding of Rad23 by the proteasomal ATPases, protecting Rad23 from degradation.

Increased stabilization may also stem from reduced proteasomal degradation. During aging, a unique frame shift mechanism generates a mutant ubiquitin molecule termed UBB (+1). UBB (+1) caps existing substrate-free ubiquitin chains and is a natural inhibitor of the 26S proteasome. Thus, by inhibiting the proteasome, endogenous substrates exhibit increased stabilization. Ubb (+1) chains may also interfere with the activity of protein kinases that are regulated by ubiquitination. Ubb (+1) was shown to be intimately related to the development of Alzheimer’s disease, where it is linked to protein aggregation [77,78,79,80,81].

While the aberrant activity of the proteasome affecting proteostasis is discussed in depth by Staller et al. in this volume, it should be noted that protein stabilization may be due to mutations in genes coding to proteasome subunits. Collectively, these mutations are the molecular basis for proteasome-associated auto-inflammatory syndromes (PRAAS). One example of such syndrome, which results from a mutation in the proteasome β subunit type 8, is CANDLE syndrome (chronic atypical neutrophilic dermatosis with lipodystrophy and elevated temperature) [82,83]. Moreover, reduced global protein clearance due to impaired proteasome activity is observed upon aging and results in the global accumulation of damaged proteins [84,85,86].

## 5. Ubiquitin-Dependent Oncoprotein Stabilization

Over the lifetime of a cell, the stability of a given protein is actively and transiently increased by post-translational modifications, of which phosphorylation is one of the more prominent. Stabilizing phosphorylations convey signals from both the extracellular and intracellular environments, granting a given protein or group of proteins enhanced activity for a limited time. P53 is stabilized and its transcriptional activity is enhanced by cellular stress signals such as UV or ionizing radiation. Such stress signals lead to P53 phosphorylation and stabilization by diverse kinases such as ATM (mutated in ataxia-telangiectasia), ATR (A-T and Rad3-related), the checkpoint kinases (Chk1 and Chk2), as well as Jun NH2-terminal kinase (JNK), p38, and others. Among the most phosphorylated sites that lead to stabilization of human P53 is Ser 15 [87,88,89,90,91,92].

In the case of multiple oncoproteins, a priming phosphorylation, which is mediated by growth-promoting signaling and mitogenic kinases such as Akt and Ras/MAPK, first stabilizes and enhances their activity. These priming phosphorylations subsequently enable the recruitment of glycogen synthase kinase-3β (GSK3-β) that catalyzes the second destabilizing phosphorylation. One well-studied case is the phosphorylation of Ser62 of c-Myc by the Ras/MAPK pathway that primes for the secondary destabilizing phosphorylation by GSK3β at Thr58, enabling the binding of the E3 ligase receptor subunit FbXW7. Phosphorylation of Ser45 of β-catenin by casein-kinase-I (CKI) is likewise stabilizing [93]. Similar stabilizing and degradation-priming phosphorylations have been observed in all FbXW7 substrates including CycE, c-Jun, Notch intracellular domain (NICD), and PGC1α [40,94,95,96].

How is the transient phosphorylation translated into protein stabilization? Recent studies from our laboratory suggest that once such oncoproteins are initially phosphorylated, they are recognized by a ubiquitin-dependent pathway that catalyzes a-typical polyubiquitin chains leading to their stabilization (see below). The ubiquitin ligase RNF4 is the central E3 enzyme in this pathway [11,97,98]. RNF4 is a RING-type ubiquitin ligase that belongs to a small group of SUMO-targeted ubiquitin ligases (STUbL). RNF4 binds to SUMOylated proteins via its four SUMO-interacting motifs (SIMs) and targets them for ubiquitination via its RING domain, connecting the ubiquitin with the SUMO pathways [99,100,101]. STUbLs are conserved from yeast to humans. The *Drosophila* genome codes for a single STUbL gene, *degringolade* (*dgrn*) [102,103], while the human genome encodes for two STUbL proteins, RNF4 and RNF111.

RNF4 and Dgrn are present in both the nucleus and the cytoplasm. Remarkably, during early *Drosophila* embryogenesis, Dgrn localization dramatically alternates between the cytoplasm to the nucleus in an embryonic cycle-regulated manner [103]. Likewise, RNF4 is localized to the cytoplasm, along the secretory pathway and the nucleus. Indeed, RNF4 substrates include the predominantly cytoplasmic translation initiation factor eIF2α, the transmembrane protein CFTR, and nuclear oncoproteins.

RNF4 and Dgrn, but not RNF111, harbor an arginine-rich region (ARM) that is the docking site for the phosphorylated oncoproteins. STUbL also contains a nucleosome-targeting motif that is required for binding to chromatin [100,104]. RNF4 regulates diverse cellular processes and serves as a transcriptional co-activator, a function that may involve CpG demetylation [105,106,107,108]. RNF4 is also required for the DNA damage response and nuclear protein quality control, both of which are topics that are beyond the scope of this review (for example see [109,110,111]; and reviewed in [112,113]).

In cancer, RNF4 has both tumor-suppressive as well as tumor-promoting activities. The cancer suppressive activities of RNF4 are SUMOylation dependent, while its cancer- promoting activities involving protein stabilization are independent of de-novo SUMOylation. Stabilization takes place in cells in which the SUMO E1 subunits SAE2 was inactivated, as was well observed upon expression of RNF4 that lacks the SIM domains.

Table 1 presents examples of SUMO-dependent and independent substrates of RNF4. A tumor-suppressive role for RNF4 was discovered in acute promyelocytic leukemia (APL), in which the oncogenic driver is a fusion protein that combines the promyelocytic leukemia protein (PML) with RARα (PML-RARα). Arsenic or retinoic acid treatment induces the SUMOylation of PML-RARα. The SUMOylated oncoprotein is bound and ubiquitinated by RNF4 and is subsequently degraded, resulting in differentiation of the tumor cells [114,115]. Remarkably, a short course of treatment with arsenic forms the basis of the current therapy and cure for APL [116].

In epithelial cancers, melanoma, and osteosarcoma, RNF4 has a pro-tumorigenic function, which is directly linked to its protein-stabilizing activity [97,98]. RNF4 binds via its ARM domain to selected phospho-oncoproteins that all share a stabilizing phosphorylation mediated by mitogenic/stress kinases. Examples are the binding of RNF4 to c-Myc upon phosphorylation of Ser62 or to β-catenin upon phosphorylation of Ser45.

Importantly, RNF4 acts upstream to the secondary destabilizing phosphorylations and the ubiquitin enzymes involved in the physiological degradation of these oncogenic substrates (e.g., E2 and E3). For example, it stabilizes cancer-mutated Myc^T58A^ and β-cateinin^S33A^ and its stabilizing activity is, accordingly, independent of FbXW7 and the E2s involved in c-Myc degradation.

## 6. Heterotypic Ubiquitin Chains Mediate Protein Stabilization

The exact molecular details involved in RNF4-dependent protein stabilization are still not fully clear. Nevertheless, molecular analysis suggests that it requires the catalysis of unique polyubiquitin chains on RNF4-stabilized substrates. Ubiquitin is a 76 amino-acid long protein that contains seven internal lysine residues (K6, K11, K27, K29, K33, K48, K63). These lysines enable the covalent linking of additional ubiquitin molecules to yield polyubiquitin chains [117,118,119]. Linkage-specific ubiquitin chains have distinct physiological outcomes; polyubiquitin chains in which all ubiquitin molecules are connected through the same internal lysine are called homotypic chains. K48-linked homotypic ubiquitin chains are involved in targeting proteins for proteasomal degradation, while K63-chains are involved in immune signaling cascades and serve as an inoculation/docking platform [120,121]. Polyubiquitin chains in which the expanding ubiquitin polymer is linked internally through diverse linkages are referred to as heterotypic or mixed chains. While the full physiological function of heterotypic ubiquitin chains (Ub^Het^) is only starting to unfold, several reports indicate that Ub^Het^ chains promote protein stabilization [122].

In the case of RNF4, the catalysis of poly-Ub^Het^ chains by RNF4 is critical for substrate stabilization. These chains are characterized by K11 and K33 internal linkages on these substrates and enhance their stability. Interestingly, it was shown that in the case of c-Myc, phosphorylation of Ser62 is required not only for RNF4-dependent stabilization but also for its association with lamins, an association that may be linked to the formation of Ub^het^ chains and to stabilization [16].

The formation of poly-Ub^Het^ chains is a shared property of other stabilized proteins. As mentioned above, EDD-dependent stabilization of β-catenin requires the catalysis of K11- and K29-internally linked Ub^Het^ chains [34]. Another example is the case of Ub^Het^ chains that are catalyzed by the virus-encoded E3 ligase mLena, which stabilizes c-Myc independently of the phosphorylations that are associated with its turnover [123]. Self-ubiquitination of Ub^Het^ chains is likewise catalyzed by RING1B, protecting them from degradation, a self-ubiquitination that is required for H2B ubiquitination and gene activation [124]. Ub^Het^ chains are also catalyzed by the E3 TRAF6, stabilizing the mutant DJ-1 and α-synuclein proteins, which are localized to Lewy bodies in sporadic Parkinson’s disease brains [125]. Ub^Het^ chains were also shown to regulate cell cycle progression, in part by stabilizing p27^Kip1^/CDKN1B, a ubiquitination that is mediated by UBCH7/UBE2L3 enzymes [126].

While our focus in this review is on stabilizing E3 ubiquitin ligases, it is likely that the identity of the ubiquitin-conjugating enzyme (E2, UbC), which pairs with the stabilizing E3, also determines the nature of the internal linkage and the type of chain formed, in turn, affecting protein stability. Moreover, it is not yet clear whether Ub^Het^ chains confer stability passively, as they are poor substrates for the proteasome, or whether they are recognized by a yet-to-be-identified active stabilization machinery. Furthermore, while the activity of the ubiquitination machinery may be intact, increased stabilization of oncoproteins may result from increased protein deubiquitination by deubiquitinase (DUBs). The role of DUBs in protein stabilization, development, and in cancer, are the focus of reviews by Diefenbacher and Mohan in this volume.

## 7. Ubiquitin-Dependent Stabilization Is Evolutionarily Conserved

Much of what we have learned regarding RNF4 and ubiquitin-dependent protein stabilization was first observed in the fly. The *Drosophila* genome codes for a single Sumo-targeted ubiquitin ligase (STUbL) enzyme ortholog to RNF4 that we termed *degringolade* (*dgrn* CG10981). As with RNF4, Dgrn is required for Wnt signaling in the developing embryo and for Notch pathway activation in the fly wing [102,103]. Likewise, in the adult *Drosophila,* Dgrn is essential for Notch pathway activation during gut regeneration upon infection, similar to its enhancement of N-ICD stability and Notch pathway activation in mouse cells [97,127]. Moreover, *dgrn* null and heterozygous mutants are immunocompromised, as Dgrn is required for the transcriptional activation of anti-microbial peptide genes downstream of the NFκB pathway, by enhancing the stability of Dif, a central immunity transcription factor [127]. Moreover, expression of Dgrn in the ovary restored fertility to *dMyc^dm1^* hypomorphic sterile females, likely by increasing the reduced dMyc protein levels in the hypomorphic mutant [Orian, unpublished]. Collectively, these data suggest that Dgrn has similar effects on protein stabilization and oncogenic pathways as RNF4, opening the door to use Dgrn as a handle, together with the power of *Drosophila* genetics, to identify novel genes within the ubiquitin-dependent stabilization pathway.

## 8. The Tumor-Promoting Activity of RNF4

In cancer, the stabilizing effect of RNF4 results in increased transcriptional activity of oncogenic transcription factors and enhances tumorigenic properties of cancer cells both in culture and in vivo. RNF4 is required for, and enhances, the transcriptional activity of Myc and β-catenin, and promotes Wnt target gene expression as well as stabilization of Notch intracellular domain protein (NICD) and Delta-dependent Notch activation [97,98].

In melanoma, the stabilizing activity of RNF4 is not limited to oncogenic transcription factors but also requires the translation initiation factor eIF2α. In non-transformed cells, phosphorylation of eIF2α on Ser51 inhibits the formation of the initiation complex and attenuates general translation. Remarkably, in cancer cells, p-eIF2α promotes oncogenic translation; phosphorylation of eIF2α in cancer cells “hijacks” the translation machinery and results in enhanced activation of tumor-promoting translation via the use of non-conventional internal ribosome entry site (IRES)-containing oncogenic mRNAs and involves unique translation machinery including proteins such as eIF5B, eIF2D, and MCT-1 that together activate tumor promoting pathways [128,129,130].

eIF2α was identified as an upstream positive regulator of RNF4-dependent gene signature (but not of the RNF4 gene itself) in human melanoma xenograft tumors conditionally expressing RNF4. In melanoma patient-derived biopsies, the levels of p-eIF2α correlate with high levels of RNF4 protein. Indeed, RNF4 binds and stabilizes p-eIF2α catalyzing Ub^het^ chains (K11, K33) similar to its stabilizing activity towards c-Myc and other oncoproteins. The phosphorylation of eIF2α is mandatory for binding, ubiquitination, and stabilization by RNF4. eIF2α is phosphorylated on Ser51 by four distinct kinases (Heme-regulated kinase HRI, PERK, GCN2, and PKR) [131], yet the identity of the kinase(s) that act(s) in the context of RNF4-dependent stabilization is the focus of current studies. Subsequently, stabilized p-eIF2α enhances the translation of mRNAs of oncogenes such as *c-myc* and *vegf*. However, and unlike in the classical stress response in non-transformed cells, RNF4 activity in cancer cells enhances oncogenic translation without inhibiting general translation [98].

A hallmark of BRAF-mutated melanoma is the rapid development of resistance to receptor kinase (RTK) inhibitors such as Vemurafenib and its analog PLX4032 in experimental models and patients. The expression of RNF4 and its stabilized proteins, including p-eIF2α, is higher in Vemurafenib-resistant cell lines. Along this line, we found that eIF2α is critical for the resistance to therapy induced by RNF4 in melanoma. For example, expression of RNF4 confers resistance to PLX4032 in human melanoma cells, and its conditional elimination from PLX4032-resistant tumors in vivo resulted in collapse of the tumors. The inability of RNF4-deficient PLX4032-resistant melanoma cells to form colonies was restored upon co-expression of eIF2α a function that is dependent on eIF2α phosphorylation. Thus, establishing the critical role of this phosphorylation to and RNF4 tumorigenesis in culture [98].

## 9. RNF4 Is Associated with Resistance to Therapy and Poor Prognosis in Human Cancer

High levels of RNF4 are observed in about 40% of melanoma patients and in 30% of colon cancer biopsies. Analysis of colon cancer biopsies suggests that an elevated level of RNF4 protein is observed specifically at the transition from adenoma to carcinoma. In melanoma, high levels of RNF4 are correlated with high levels of p-eIF2α and are in correlation with unresponsiveness to RTK therapy. In both melanoma and luminal type-A breast cancer, high levels of RNF4 are associated with poorer prognosis [97,98]. The importance of RNF4 in cancer is a new concept and the mechanisms by which RNF4 confers resistance to molecular therapy are still unclear. It is also unknown whether these resistance mechanisms are general, are relevant also to immune checkpoint inhibitors therapy, or are specific to RTK therapy. Additonally, the full spectrum of tumors that collapse upon loss of RNF4 is still unknown. For example, high levels of RNF4 correlate with longer survival times in lung cancer but are associated with poor prognosis in gastric cancer [132].

## 10. Implications of RNF4 and Ubiquitin-Dependent Protein Stabilization in Cancer

**Figure 2:**
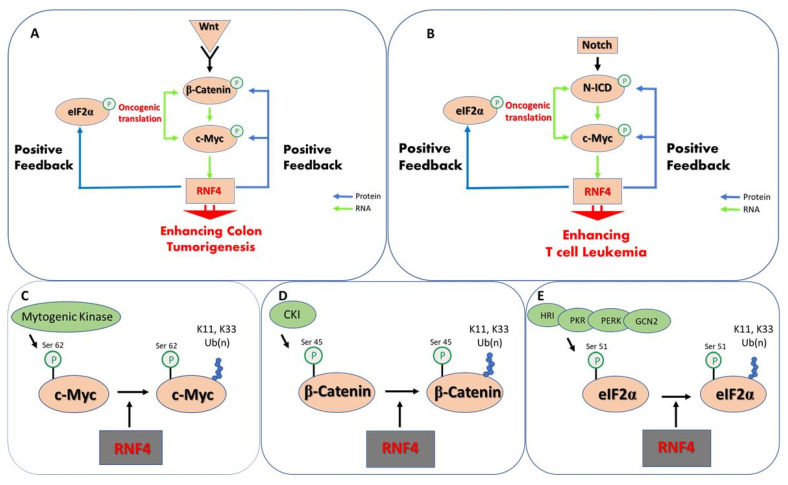
Protein stabilization is a feed-forward mechanism enhancing tumorigenesis. (**A**) In colon cancer activation of Wnt signaling results in the nuclear translocation of the β-catenin co-activator that together with TCF activates the transcription of c-Myc. c-Myc binds to the *rnf4* gene and induces its expression. RNF4 protein in turn stabilizes both p^(S45)^-β-catenin and p^(S62)^-c-Myc and enhances their stability and transcriptional activity. In parallel, RNF4 stabilizes p^(S51)^eIF2α that is central for the activation of oncogenic translation including c-Myc and VEGF. (**B**) A similar mechanism operates in acute T-cell leukemia, where Notch pathway activation induces the nuclear accumulation of Notch intracellular domain protein (N-ICD) that binds to a c-Myc enhancer inducing c-Myc expression, that subsequently increases RNF4 expression. (**C**–**E**) Molecular details of kinases and site of phosphorylation of the indicated oncoproteins that prime for RNF4-dependent K11, K33 polyubiquitination and protein stabilization.

Ubiquitin-dependent protein stabilization emerges as a positive feed-forward loop that enhances the activity of key oncoproteins and promotes oncogenic translation (Figure 2). Several of the oncogenic signaling pathways, such as Notch and Wnt, activate the transcription of c-Myc that binds to the *rnf4* gene and induces its expression [133,134]. Indeed, RNF4 expression is elevated in experimental models of Myc-driven tumors [135]. In turn, and once expressed, RNF4 stabilizes and enhances the activity of c-Myc, β-catenin, and N-ICD, enhancing the expression of pathway target genes [97]. In parallel, RNF4 enhances oncogenic translation by stabilizing p-eIF2α, including the translation of c-Myc and angiogenic factors such as VEGF [128,129].

Taken together, the accumulating data suggest that the activity of RNF4 and the ubiquitin-dependent protein stabilization pathway constitute a fundamental basis for tumorigenesis, are essential for cancer cell survival, and act as an Achilles’ heel for cancer cells. Inhibition of enzymes within this pathway will, therefore, will result in the eradication of degradation-resistant tumors, which will be highly relevant to a large yet specific group of cancer patients.

**Table 1:** Examples of RNF4 substrates. Upper table: Substrates that are recognized once SUMOylated.

**SUMO-Dependent Substrates of RNF4**	
**Protein**	**References**
PML	[114,115,116,136]
JARID1B	[137]
SP100 *	[138]
IFI16	[139]
CFTR	[140]
Pea3	[141]
BLM	[109]
FANCI, FANCD2 (ID complex)	[142]
PIAS1, PIAS2, PIAS3	[143]
ZNF451	[143]
NSMCE2	[143]
Ubc9	[143]
BRCA1–BARD1 complex	[143]
MDC1	[144]
PARIS/ZNF746	[145]
KAP1	[104]
Rta	[146]
NDRG2	[147]
Tax	[148]
Ataxin-7	[149]
FXR	[150]
**SUMO-independent substrates of RNF4**	
**Protein**	**Reference**
c-Myc	[97]
c-Jun	[97]
β-catenin	[97]
NICD	[97]
PGC1 α	[97]
eIF2α	[98]
HIF1α	[98]

“*” indicates partial proof. Lower table: Substrates that are recognized by RNF4 independent of SUMOylation.

## Data Availability

Not applicable.
